# A Global Optimization Approach to Multi-Polarity Sentiment Analysis

**DOI:** 10.1371/journal.pone.0124672

**Published:** 2015-04-24

**Authors:** Xinmiao Li, Jing Li, Yukeng Wu

**Affiliations:** School of Information Management and Engineering, Shanghai University of Finance and Economics, Shanghai, China; Beihang University, CHINA

## Abstract

Following the rapid development of social media, sentiment analysis has become an important social media mining technique. The performance of automatic sentiment analysis primarily depends on feature selection and sentiment classification. While information gain (IG) and support vector machines (SVM) are two important techniques, few studies have optimized both approaches in sentiment analysis. The effectiveness of applying a global optimization approach to sentiment analysis remains unclear. We propose a global optimization-based sentiment analysis (PSOGO-Senti) approach to improve sentiment analysis with IG for feature selection and SVM as the learning engine. The PSOGO-Senti approach utilizes a particle swarm optimization algorithm to obtain a global optimal combination of feature dimensions and parameters in the SVM. We evaluate the PSOGO-Senti model on two datasets from different fields. The experimental results showed that the PSOGO-Senti model can improve binary and multi-polarity Chinese sentiment analysis. We compared the optimal feature subset selected by PSOGO-Senti with the features in the sentiment dictionary. The results of this comparison indicated that PSOGO-Senti can effectively remove redundant and noisy features and can select a domain-specific feature subset with a higher-explanatory power for a particular sentiment analysis task. The experimental results showed that the PSOGO-Senti approach is effective and robust for sentiment analysis tasks in different domains. By comparing the improvements of two-polarity, three-polarity and five-polarity sentiment analysis results, we found that the five-polarity sentiment analysis delivered the largest improvement. The improvement of the two-polarity sentiment analysis was the smallest. We conclude that the PSOGO-Senti achieves higher improvement for a more complicated sentiment analysis task. We also compared the results of PSOGO-Senti with those of the genetic algorithm (GA) and grid search method. From the results of this comparison, we found that PSOGO-Senti is more suitable for improving a difficult multi-polarity sentiment analysis problem.

## Introduction

User-generated content (UGC) from various social media platforms has increased significantly with the rapid development of Web 2.0 technology. The abundance of UGC provides significant value for enterprise strategy formation and market forecasting. Automatic sentiment analysis is crucial to this process. For example, sentiment analysis of Twitter data can potentially be used to predict stock prices [1]. Opinions from product discussion forums and product review websites can support decision making in purchasing and the identification of new business opportunities [2–3].

Sentiment analysis is also known as emotional polarity computation [4–5], opinion extraction or semantic classification [6–7]. It is defined as the process of identifying the sentiment and opinion (e.g., negative or positive) in a given piece of text [6]. The performance of automatic sentiment analysis primarily depends on feature selection and sentiment classification.

It is both difficult and important to select the right set of features for sentiment classification. UGC in social media usually contains hundreds or thousands of words. One does not know which features are relevant for a particular sentiment classification task. One common approach is to construct a dictionary. However, dictionary construction requires significant human effort and thus is labor-intensive, expensive and time-consuming. Some public sentiment dictionaries can be used, such as the SentiWord dictionary for English sentiment analysis and the HowNet dictionary for Chinese. However, the UGC in social media differs significantly from traditional texts. First, the words and phrases used in social media are highly random and irregular. Second, many words and phrases used in social media are created by network users who are expressing their own sentiments. The creation, update and dissemination speeds of network words and phrases far exceed the update speed of a sentiment dictionary. Therefore, many words and phrases used in social media, in particular, domain-specific words and phrases, are not included in the sentiment dictionary. Thus, dictionary-based approaches usually do not produce satisfactory results for UGC sentiment analysis. Several other feature selection methods,primarily including information gain (IG), the Chi-squared test (χ^2^), document frequency (DF), and mutual information (MI), are used for UGC sentiment analysis. However, these methods produce high-dimensional feature space. Therefore, the identification of an optimal subset of features for a particular sentiment analysis task is crucial. IG has demonstrated superior performance in sentimental term selection [7–10]. Abbasi et al. studied opinion classification in web forums based on IG and SVM methods [7]. The researchers indicated that although different automated and manual approaches have been used to craft sentiment classification feature sets, little emphasis has been placed on feature subset selection techniques [7]. Gamon and Yi et al. used log likelihood to select important attributes from a large initial feature space [11–12]. Wiebe et al. evaluated the effectiveness of various potential subjective elements (PSEs) for subjectivity classification based on their occurrence distribution across classes [13]. However, many powerful techniques have not been explored [14]. Feature subset selection techniques have two important benefits [14]. First, they can potentially improve classification accuracy and efficiency. Second, such techniques can remove redundant, noisy features and provide greater insight into important class attributes, resulting in a better understanding of sentiment arguments and characteristics [15–16].

Machine learning approaches have been extensively applied to sentiment analysis in recent years. The machine learning approach considers sentiment analysis as a text classification problem. Machine learning approaches include maximum entropy, the naïve Bayes algorithm, the artificial neural network (ANN), and SVM. The SVM has demonstrated superior performance, and its use is prevalent [6–7, 17–18]. Popular kernel functions of the SVM include linear, sigmoid, polynomial and radical basis function (RBF) kernels [19]. Kim et al. [20] compared four SVM kernel functions in sentiment analysis and discovered that the RBF kernel is the most effective kernel function. Chen and Tseng [21] used both linear and RBF kernels to assess the quality of user reviews. An RBF kernel contains two kernels, the penalty parameter *c* and kernel parameter *γ*. The selection of the appropriate parameters (*c*, γ) enables an SVM to achieve the optimal performance [22, 23]. If the parameter values are poorly established, the results may be unsatisfactory.

Moreover, feature subset choice influences the appropriate SVM parameters, and vice versa [24]. Therefore, the optimal feature subset and SVM parameters must be obtained simultaneously. Some of the available literature on sentiment analysis only explores feature selection techniques. However, such works do not combine feature subset and parameter selection for a particular sentiment analysis task. Therefore, a global optimization strategy for the selection of the feature subset dimension *k* and the SVM parameters *c* and γ is needed. Table 1 summarizes the automatic sentiment analysis studies that utilized SVM. We present each study by analyzing their (1) optimal feature subset selection method, (2) SVM parameter optimization and (3) global optimization (feature subset and SVM parameters optimization). From Table 1, some researchers have explored feature subset selection approaches. In addition, some studied SVM parameter optimization approaches. However, in the sentiment analysis research, little emphasis has been placed on a global optimization approach for the selection of the optimal feature subset and SVM parameters.

**Table 1:** SVM-based Sentiment Analysis Research Summary.

Research	Feature Subset Selection	SVM Parameters Optimization	Global Optimization
Desmet (2013) [25]	No	No	No
Moraes (2013) [26]	No	No	No
Basari (2013) [27]	No	Yes, PSO	No
Lane (2012) [28]	Yes, Chi-square approach and taking the top 250 features	Yes, Manual setting	No
Abbasi et al. (2010) [29]	No	No	No
Li and Wu (2010) [30]	No	No	No
Abbasi et al. (2008) [7]	No	No	No
Tan and Zhang (2008) [8]	Yes, Predefined size of feature set	No	No
Coussement and Poel (2008) [22]	No	Yes, Grid search approach	No
Xia (2008) [31]	Yes, Chi-square approach and remaining the top 60% sentiment words	No	No
Pang (2002) [6]	No	No	No

Some research has explored the global optimization approach in other fields [32]. For example, to recognize the presence of a power disturbance and classify an existing disturbance into a particular type, Manimala et al. [33] used the GA approach for feature selection and parameter optimization. Huang et al. [34] proposed a GA-SVM credit scoring model. This model used a GA to perform simultaneous feature selection tasks and model parameter optimization processes. Huang [35] presented a GA-SVR model for effective stock selection based on the support vector regression (SVR) and GA methods. However, at present, the effectiveness of applying a global optimization approach to sentiment analysis remains unclear.

To address this gap, we propose a global optimization-based sentiment analysis (PSOGO-Senti) approach to improve sentiment analysis with IG for feature selection and SVM as the learning engine. The PSOGO-Senti approach utilizes a particle swarm optimization algorithm to obtain a global optimal combination of feature subset dimension *k* and parameters *c* and γ in the SVM. We raise the following research questions. (1) What is the effectiveness of a global optimization-based sentiment analysis (PSOGO-Senti) approach in sentiment analysis? Is the PSOGO-Senti approach effective and robust for sentiment analysis tasks in different domains? (2) To what extent can the PSOGO-Senti approach improve the sentiment analysis results for two-polarity and multi-polarity cases? (3) What are the differences between the optimal feature subset selected by PSOGO-Senti and the features in the sentiment dictionary for domain-specific sentiment analysis tasks? (4) What are the differences between the results of PSOGO-Senti, the grid search method (GSM) and GA for two-polarity and multi-polarity domain-specific sentiment analysis tasks?

The remainder of this paper is organized as follows. First, we review the methods developed in the sentiment analysis field with a focus on IG and SVMs. Next, we discuss the challenges associated with UGC sentiment analysis and propose the PSOGO-Senti approach. Section 2 provides an overview of our proposed PSOGO-Senti approach and explains its components. Section 3 presents the experimental evaluations. We conclude the paper with details regarding our research contributions and future studies.

## Proposed Approach: PSO-based Global Optimization for Sentiment Analysis (PSOGO-Senti)

**Fig 1:**
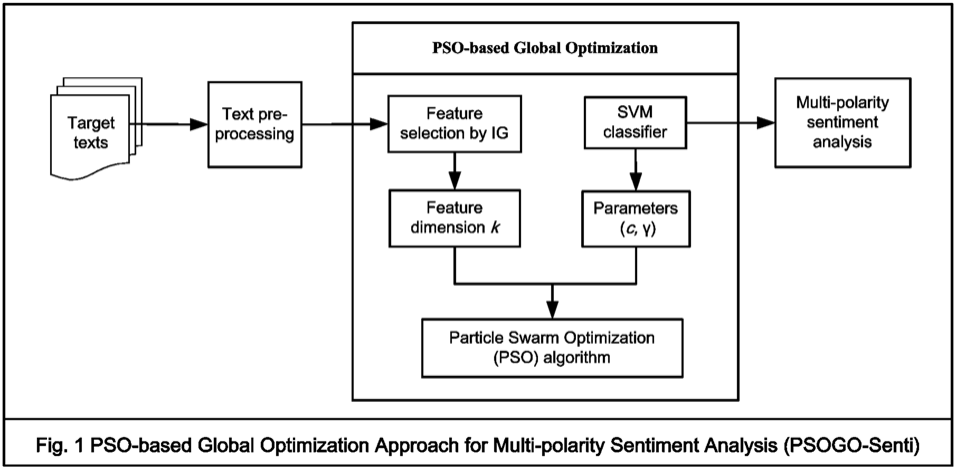
PSO-based Global Optimization Approach for Multi-Polarity Sentiment Analysis (PSOGO-Senti).

To address the research questions, we designed the PSOGO-Senti model for automatic sentiment identification with IG for feature selection and SVM as the learning engine, as shown in Fig 1. The PSOGO-Senti approach utilizes a PSO algorithm to obtain a global optimal combination of the feature dimension *k* and the parameters *c* and γ in the SVM. The goal is to construct a model that classifies target text into multi-level polarities. First, training texts are collected and parsed. Text pre-processing includes actions such as indexing, part-of-speech (POS) tagging and stop word removal. Second, an initial term matrix is produced by extracting terms from the target text. Third, IG is used to select features from the initial term matrix. After feature selection, a new term matrix is produced and fed into the SVM. Finally, we estimate the optimal feature dimension and the optimal classifier parameters through the PSO algorithm. The final optimized SVM model is used to create the polarity classifier. We describe each component in this section.

### Text Pre-processing

Text pre-processing is crucial to text classification. Prior to analysis, the text processing component is designed to index and extract the terms that are employed in subsequent classification steps. Words that do not exhibit useful meanings are considered stop words and are removed to optimize performance. To perform sentiment analysis of Chinese texts, word segmentation must be performed because Chinese does not contain word boundaries. Based on textual content, the extracted terms should be representative of the original texts.

### Feature Selection by IG

The objective of feature selection is to select a group of informative features that can retain the majority of information concerning the original data and that can generate the best prediction performance [36]. Our adopted feature selection method is IG.

The discrimination ability of a feature is measured by the IG, which is based on Shannon entropy [37]. The entropy of the class distribution *H*(*C*) is defined as $H(C)=-\sum_{i=1}^{n}P(C_{i})•\log_{2}P(C_{i})$, where *P*(*C*_*i*_) denotes the probability that a review does not belong to class *C*_*i*_. The *IG*(*F*) of a feature *f* describes the difference in the entropy of the class distribution *H*(*C*) and the additional amount of information provided by the feature of the class, which is noted as the conditional entropy *H*(*C* | *F*). *H*(*C* | *F*) is defined as $H(C|F)=-P(f)\sum_{i=1}^{n}P(C_{i}|f)\log_{2}P(C_{i}|f)-P(\bar{f})\sum_{i=1}^{n}P(C_{i}|\bar{f})\log_{2}P(C_{i}|\bar{f})$, where *P*(*f*) denotes the probability that a review contains feature *f*, $P(\bar{f})$ denotes the probability that a review does not contain feature *f*, *P*(*C*_*i*_ | *F*) denotes the probability that a review belongs to class *C*_*i*_ with the condition that the review contains feature *f*, and $P(C_{i}|\bar{f})$ denotes the probability that a review belongs to class *C*_*i*_ with the condition that the review does not contain feature *f* The IG is defined as *IG*(*F*) = *H*(*C*) - *H*(*C* | *F*)

### SVMs

Text classification can be performed by adopting various machine learning algorithms, such as naïve Bayes, K-nearest neighbor (KNN), a neural network and an SVM. The SVM has shown superior performance in sentiment analysis compared to the other aforementioned algorithms because it overcomes difficulties such as the dimensionality curse, local minimization and overfitting problems. By defining different kernel functions, an SVM can realize various learning algorithms, such as polynomial approximation, RBF and multilayer perceptron neural network (MLPNN). The SVM has been extensively used in various fields due to its outstanding classification and generalization capability. The performance of an SVM is dependent on the kernel function. Kernel function selection is a crucial step when handling learning tasks with SVMs. Although different kernel functions can be tested in the future, we focus on one of the most extensively utilized kernels in this study, namely, the RBF.

The RBF has two parameters: the penalty parameter *c* and core parameter γ. RBF parameter estimation is closely related to the performance of an SVM classifier. Vapnik et al. [38] suggested that although different kernel functions achieve similar performances, the penalty parameter *c* and core parameter γ are the key factors in the performance of an SVM. In addition to the selection of *k* during feature dimension selection, parameter estimation is another important component of an SVM classifier.

### PSO

PSO is an efficient evolutionary computation learning algorithm. The concept of PSO is designed to simulate social behavior based on information exchange and is intended for practical applications. Within the problem space, each potential solution can be considered as a particle in a swarm. Every particle with a certain velocity can adjust its directional path according to its own flight experience and the flight experiences of its companions. This superior strategy effectively mines the optimal regions of complex search spaces via the interaction of individuals in a population of particles [39].

The PSO algorithm can be divided into four steps within a process period [39–40]. First, particles are initialized in a population of random solutions. Second, each particle obtains its *pbest*_*j*_ by comparing its current fitness to the fitness of its previous position, where *pbest*_*j*_ is the position of the *j* th particle with the highest fitness value at a given iteration. It can be regarded as the best solution in terms of the *j* th particle. Third, the *gbest* for all particles in the population is determined. The best position for all *pbest* particles is referred to as the global best *gbest* and can be regarded as the best solution for all particles. Finally, the PSO algorithm executes a search for the optimal solutions by updating the generations. In each generation, the position and velocity of the *j* th particle are updated with the *pbest*_*j*_ and *gbest* of the swarm population. The update equations can be formulated as

$$v_{j}^{}(t+1)=\omega v_{j}^{}(t)+d_{1}(pbest_{j}-x_{j}^{}(t))+d_{2}(gbest-x_{j}^{}(t))$$ (1)

$$x_{j}^{}(t+1)=x_{j}^{}(t)+v_{j}^{}(t+1)$$ (2)

where ω is the inertia weight, *v*_*j*_ is the velocity, *x*_*j*_ is the particle position, and *d*_1_ and *d*_2_ are learning factors.

In our PSOGO-Senti model, we need to run the PSO algorithm to obtain the following optimized parameters: the feature subset dimension *k*, SVM penalty parameter *c* and core parameter γ. We select the best combination of (*k*, *c*, *γ*) to construct the final PSOGO-Senti model.

## Experimental Design

To address our research questions, we conducted three experiments on two datasets. In experiment 1, PSOGO-Senti is used to improve two-polarity, three-polarity and five-polarity sentiment analysis on a dataset of Ctrip tourism reviews. In experiment 2, PSOGO-Senti is used to improve two-polarity, three-polarity and five-polarity sentiment analysis on a dataset of Guahao medical reviews. In experiment 3, we compare the results of PSOGO-Senti with those of the GSM- and GA-baesd approaches. The results are presented here.

### Data

In recent years, with the improvement of living standards and the rapid development of social media services, people have come to pay more attention to health and tourism. Chinese-based UGC in social media that relates to health and travel is expanding. People are more likely to obtain health information from and share their health experiences on such social media websites [41]. Sentiment analysis on medical reviews is very important for patients to make better health decisions. In the tourism field, tourists check opinions and experiences published by other travelers on different websites when planning their own vacations. On the other hand, the vast amount of information available publicly on the Web could help tourism companies and related organizations conduct polls and market research. Thus, sentiment analysis on tourism reviews is very crucial for both tourists and tourism companies and organizations. In this research, we selected two Chinese UGC datasets as the experimental data used to demonstrate the performance of PSOGO-Senti. One is a dataset of Ctrip tourism reviews, and the other is a dataset of Guahao medical reviews. We conducted experiments for two-polarity, three-polarity and five-polarity Chinese sentiment analyses.

We employed the Chinese reviews of the tourist zone from http://you.ctrip.com/place/. Ctrip.com is the most popular tourism review website in China. We randomly selected 5,000 reviews from the website. The review ratings on this website range from 1 to 5. One-thousand reviews were selected for each rating scale. For the two-polarity sentiment analysis experiments, we consider 1–2 to be negative and 4–5 to be positive. Neutral reviews with ratings of 3 are excluded from the dataset. For the three-polarity sentiment analysis experiments, we consider 1–2 to be negative, 3 to be neutral, and 4–5 to be positive. For the five-polarity sentiment analysis experiments, we consider 1 to be strongly negative, 2 to be negative, 3 to be neutral, 4 to be positive, and 5 to be strongly positive. We perform a 10-fold cross validation to estimate the optimized parameters.

The Guahao dataset is from http://www.guahao.com/search/experts/. Guahao.com is the most popular health consultation and medical guidance platform in China. This platform hosts a large number of comments on doctors from more than 3900 hospitals distributed within 31 provinces and municipalities in China. We randomly selected 5,000 reviews from the platform. The review ratings range from 1 to 5 on Guahao.com. One-thousand reviews were selected for each rating scale. For the two-polarity sentiment analysis experiments, we consider 1–2 to be negative and 4–5 to be positive. Neutral reviews with ratings of 3 are excluded from the dataset. For the three-polarity sentiment analysis experiments, we consider 1–2 to be negative, 3 to be neutral, and 4–5 to be positive. For the five-polarity sentiment analysis experiments, we consider 1 to be strongly negative, 2 to be negative, 3 to be neutral, 4 to be positive, and 5 to be strongly positive.

### Evaluation Metrics

To evaluate the performance of the sentiment analysis, we adopted several standard metrics, recall, precision and the F-measure, which were employed in previous studies [7]. These metrics are defined as follows:

$$R_{i}=\frac{\text{Number of documents belonging to class i }}{\text{Number of documents belonging to class i before classification}}\times 100\%$$ (3)

$$P_{i}=\frac{\text{Number of documents belonging to class i }}{\text{Number of documents belonging to class i after classification}}\times 100\%$$ (4)

$$\bar{R}=\frac{\sum_{i=1}^{n}R_{i}}{N}\times 100\%$$ (5)

$$\bar{P}=\frac{\sum_{i=1}^{n}P_{i}}{N}\times 100\%$$ (6)

$$F-measure=\frac{2\bar{P}\bar{R}}{\bar{P}+\bar{R}}$$ (7)

where *R*_*i*_ is the retrieval recall ratio of polarity *i*, *p*_*i*_ is the precision, $\bar{R}$ is the average recall ratio, $\bar{P}$ is the average precision, and *N* is the number of classes.

### Experimental Results and Analysis

#### Experiment 1: Ctrip data

To answer the research questions, we compared the PSOGO-Senti performance with several benchmarks to assess the improvement of global optimization over simple optimization strategies: (1) the optimal feature dimension *k* and default SVM parameters *c* and γ (*c* = 1, γ = 0.1); (2) the optimal feature dimension *k* and optimized SVM parameter γ with the default parameter *c* (*c* = 1); (3) the optimal feature dimension *k* and optimized SVM parameter *c* with the default parameter γ (γ = 0.1); (4) randomly select 5,000 as the feature dimension with the optimized SVM parameter *c* and default parameter γ (γ = 0.1); (5) randomly select 5,000 as the feature dimension with the optimized SVM parameters *c* and γ; (6) randomly select 5,000 as the feature dimension with the optimized SVM parameter γ and default parameter *c* (*c* = 1); (7) randomly select 5,000 as the feature dimension with a default SVM parameter setting, where *c* = 1 and γ = 0.1. We present a 10-fold cross-validation performance of the two-polarity sentiment analysis in Table 2.

**Table 2:** Performance Comparison of PSOGO-Senti and Benchmarks in the Two-Polarity Sentiment Analysis (Ctrip Dataset).

	Precision	Recall	F-measure
**Benchmark 1**: Optimal *k* + Default *c* + Default γ	0.903	0.903	0.903
**Benchmark 2**: Optimal *k* + Default *c* + Optimal γ	0.788	0.784	0.784
**Benchmark 3**: Optimal *k* + Optimal *c* + Default γ	0.905	0.905	0.905
**Benchmark 4**: *k* = *5000* + Optimal *c* + Default γ	0.89	0.89	0.89
**Benchmark 5**: *k* = *5000* + Optimal *c* + Optimal γ	0.891	0.891	0.891
**Benchmark 6**: *k* = *5000* + Default *c* + Optimal γ	0.838	0.832	0.829
**Benchmark 7**: *k* = *5000* + Default *c* + Default γ	0.87	0.87	0.87
**PSOGO-Senti:**	**0.906**	**0.906**	**0.906**

The performance of the two-polarity sentiment analysis reveals several interesting findings. First, we observed that the PSOGO-Senti model achieved the best performance with an average precision of 0.906, average recall of 0.906 and F-measure of 0.906. These results indicated that the PSOGO-Senti model achieved satisfactory performance in the two-polarity sentiment analysis on the dataset of Ctrip tourism reviews. Second, from the performance comparison between PSOGO-Senti and benchmark 7, we found that the PSOGO-Senti approach improved over the random IG subset dimension *k* and default SVM parameters *c* and γ approach by 4.138%. From the performance comparison between PSOGO-Senti and benchmark 1, we found that the PSOGO-Senti approach improved the two-polarity sentiment analysis by 0.332%. From the performance comparison between PSOGO-Senti and benchmark 5, we found that the PSOGO-Senti approach improved the performance by 1.684%. From the performance comparison between PSOGO-Senti and benchmark 6, we found that the PSOGO-Senti approach improved sentiment analysis by 9.288%. From the performance comparison between PSOGO-Senti and benchmark 4, we found that the PSOGO-Senti approach improved the performance by 1.798%. The comparison of PSOGO-Senti and benchmark 2 revealed that the PSOGO-Senti model improved the performance of the sentiment analysis by 15.561%. The comparison of PSOGO-Senti and benchmark 3 revealed that the PSOGO-Senti model improved the performance of the sentiment analysis by 0.110%.

The performance of the three-polarity sentiment analysis is shown in Table 3. Several interesting findings arose as a result of this analysis. First, we observed that the PSOGO-Senti model achieved the best performance with an average precision of 0.695, average recall of 0.696 and F-measure of 0.695. Second, from the performance comparison between PSOGO-Senti and benchmark 7, we found that the PSOGO-Senti model improved over the random IG subset dimension *k* and default SVM parameters *c* and γ approach by 6.595%. The comparison of PSOGO-Senti and benchmark 1 indicated that the PSOGO-Senti model improved the performance of the sentiment analysis by 0.579%. From the performance comparison between PSOGO-Senti and benchmark 5, we found that the PSOGO-Senti model improved the performance of the three-polarity sentiment analysis by 1.312%. The comparison of PSOGO-Senti and benchmark 6 revealed that the PSOGO-Senti model improved the performance of the sentiment analysis by 15.066%. The comparison of PSOGO-Senti and benchmark 4 revealed that the PSOGO-Senti model improved the performance of the sentiment analysis by 1.608%. The comparison of PSOGO-Senti and benchmark 2 indicated that the PSOGO-Senti model improved the performance of the sentiment analysis by 15.257%. The performance comparison of PSOGO-Senti and benchmark 3 revealed that the PSOGO-Senti model improved the three-polarity sentiment analysis by 0.289%.

**Table 3:** Performance Comparison of PSOGO-Senti and Benchmarks in the Three-Polarity Sentiment Analysis (Ctrip Dataset).

	Precision	Recall	F-measure
**Benchmark 1**: Optimal *k* + Default *c* + Default γ	0.692	0.692	0.691
**Benchmark 2**: Optimal *k* + Default *c* + Optimal γ	0.614	0.611	0.603
**Benchmark 3**: Optimal *k* + Optimal *c* + Default γ	0.693	0.694	0.693
**Benchmark 4**: *k* = *5000* + Optimal *c* + Default γ	0.683	0.687	0.684
**Benchmark 5**: *k* = *5000* + Optimal *c* + Optimal γ	0.685	0.689	0.686
**Benchmark 6**: *k* = *5000* + Default *c* + Optimal γ	0.623	0.616	0.604
**Benchmark 7**: *k* = *5000* + Default *c* + Default γ	0.654	0.662	0.652
**PSOGO-Senti:**	**0.695**	**0.696**	**0.695**

The performance of the five-polarity sentiment analysis is detailed in Table 4. Several interesting findings were observed as a result of this analysis. First, we observed that the PSOGO-Senti model achieved the best performance with an average precision of 0.521, average recall of 0.522 and F-measure of 0.519. Second, from the performance comparison between PSOGO-Senti and benchmark 7, we found that the PSOGO-Senti model improved over the random IG subset dimension *k* and default SVM parameters *c* and γ by 14.066%. The comparison of PSOGO-Senti and benchmark 1 revealed that the PSOGO-Senti model improved the performance of the sentiment analysis by 1.170%. The comparison of PSOGO-Senti and benchmark 5 revealed that the PSOGO-Senti model improved the performance of the sentiment analysis by 0.581%. The comparison of PSOGO-Senti and benchmark 6 indicated that the PSOGO-Senti model improved the performance of the sentiment analysis by 61.682%. The comparison of PSOGO-Senti and benchmark 4 revealed that the PSOGO-Senti model improved the performance of the sentiment analysis by 3.800%. The comparison of PSOGO-Senti and benchmark 2 indicated that the PSOGO-Senti model improved the performance of the sentiment analysis by 11.613%. The comparison of PSOGO-Senti and benchmark 3 indicated that the PSOGO-Senti model improved the performance of the sentiment analysis by 0.193%.

**Table 4:** Performance Comparison of PSOGO-Senti and Benchmarks in the Five-Polarity Sentiment Analysis (Ctrip Dataset).

	Precision	Recall	F-measure
**Benchmark 1**: Optimal *k* + Default *c* + Default γ	0.516	0.517	0.513
**Benchmark 2**: Optimal *k* + Default *c* + Optimal γ	0.473	0.482	0.465
**Benchmark 3**: Optimal *k* + Optimal *c* + Default γ	0.521	0.522	0.518
**Benchmark 4**: *k* = *5000* + Optimal *c* + Default γ	0.509	0.509	0.500
**Benchmark 5**: *k* = *5000* + Optimal *c* + Optimal γ	0.517	0.519	0.516
**Benchmark 6**: *k* = *5000* + Default *c* + Optimal γ	0.479	0.400	0.321
**Benchmark 7**: *k* = *5000* + Default *c* + Default γ	0.474	0.480	0.455
**PSOGO-Senti:**	**0.521**	**0.522**	**0.519**

Based on the dataset of Ctrip tourism reviews, from the comparison between the feature subset optimized by the PSOGO-Senti model and features in HowNet, which is the most popular Chinese sentiment dictionary, we observed several interesting findings. First, we found that the features in the optimal feature subset selected by PSOGO-Senti were far fewer than those in HowNet. The results in Table 5 showed that there are 8742 features in HowNet. There are 1120 features in the optimal feature subset selected by PSOGO-Senti in the two-polarity sentiment analysis. In the three-polarity sentiment analysis, there are 1074 features in the optimal feature subset. There are 5000 features in the optimal feature subset selected by PSOGO-Senti in the five-polarity sentiment analysis. The results indicated that PSOGO-Senti can effectively remove redundant and noisy features. Second, in the feature subset optimized by PSOGO-Senti, a high proportion of the features are not included in the HowNet dictionary. The extracted unique features in the optimal feature subset are usually valuable, domain-specific and possess a high-explanatory power, such as “商业化” (commercialization), “流氓” (rogue), “假货” (fake goods), “黑店” (gangster inn), “流连忘返” (linger on and forget to return), “不虚此行” (the trip has been well worthwhile), and “世外桃源” (wonderland). Herein, the explanatory power is defined as the ability to differentiate the various sentiment categories of UGC reviews. In the two-polarity sentiment analysis, 927 features are in the optimal feature subset of PSOGO-Senti, but not in the HowNet dictionary. That is to say, 82.77% of the features in the optimal feature subset are not included in the HowNet dictionary. In the three-polarity sentiment analysis, 888 features are in the optimal feature subset of PSOGO-Senti, but not in HowNet. That is to say, 82.68% of the features in the optimal feature subset are not included in HowNet. In the five-polarity sentiment analysis, 4363 features are in the optimal feature subset of PSOGO-Senti, but not in HowNet. That is to say, 87.26% of the features in the optimal feature subset are not included in HowNet. Table 6 lists certain features that are in the optimal feature subset of PSOGO-Senti but not in the HowNet dictionary. This indicates that PSOGO-Senti can extract and select an optimal feature subset with a higher-explanatory power for tourism-specific sentiment analysis. Based on the optimal feature subset, PSOGO-Senti achieves satisfactory performance in tourism sentiment analysis.

**Table 5:** Comparison between the Optimal Feature Subset Selected by PSOGO-Senti and the HowNet Dictionary.

HowNet Dictionary	Optimal feature subset dimension	8742					
**PSOGO-Senti**	Dataset	Ctrip Dataset			Guahao Dataset		
	Polarities	Two polarity	Three polarity	Five polarity	Two polarity	Three polarity	Five polarity
	Optimal feature subset dimension	1120	1074	5000	2537	762	1686
	Features in PSOGO-Senti but not in HowNet	927	888	4363	2166	624	1403
	The percentage of features in PSOGO-Senti but not in HowNet	82.77%	82.68%	87.26%	85.38%	81.89%	83.21%

**Table 6:** Some of the Features in the Optimal Feature Subset of PSOGO-Senti but not in the HowNet Dictionary (Ctrip Dataset).

	Features
Verb	忽悠 (hoodwink), 恐吓 (threaten), 上当 (be fooled), 受骗 (be cheated), 宰 (swindle money out of customers), 坑人 (harm), 拉客 (soliciting), 享受 (enjoy)
Adjective	般般 (so so), 凑合 (make do in a bad situation), 标志性 (landmark)
Noun	流氓 (rogue), 假货 (fake goods), 黑店 (gangster inn), 垃圾 (shit), 商业化 (commercialization), 天堂(paradise)
Phrase	豁然开朗 (be suddenly enlightened), 流连忘返 (linger on and forget to return), 不虚此行(the trip has been well worthwhile), 世外桃源 (wonderland)

#### Experiment 2: Guahao data

Similar to experiment 1, we conduced experiment 2 based on the Guahao data. We used the same benchmark settings as those in experiment 1. We present a 10-fold cross-validation performance of the two-polarity sentiment analysis in Table 7.

**Table 7:** Performance Comparison of PSOGO-Senti and Benchmarks in the Two-Polarity Sentiment Analysis (Guahao Dataset).

	Precision	Recall	F-measure
**Benchmark 1**: Optimal *k* + Default *c* + Default γ	0.878	0.878	0.877
**Benchmark 2**: Optimal *k* + Default *c* + Optimal γ	0.901	0.899	0.899
**Benchmark 3**: Optimal *k* + Optimal *c* + Default γ	0.921	0.921	0.921
**Benchmark 4**: *k* = *5000* + Optimal *c* + Default γ	0.920	0.920	0.920
**Benchmark 5**: *k* = *5000* + Optimal *c* + Optimal γ	0.921	0.921	0.921
**Benchmark 6**: *k* = *5000* + Default *c* + Optimal γ	0.856	0.842	0.841
**Benchmark 7**: *k* = *5000* + Default *c* + Default γ	0.899	0.898	0.898
**PSOGO-Senti:**	0.922	0.922	0.922

The performance of the two-polarity sentiment analysis reveals several interesting findings. First, we observed that the PSOGO-Senti model achieved the best performance with an average precision of 0.922, average recall of 0.922 and F-measure of 0.922. These results indicated that the PSOGO-Senti model achieved satisfactory performance in the two-polarity sentiment analysis on the dataset of Guahao medical reviews. Second, from the performance comparison between PSOGO-Senti and benchmark 7, we found that the PSOGO-Senti approach improved over the random IG subset dimension *k* and default SVM parameters *c* and γ approach by 2.673%. From the performance comparison between PSOGO-Senti and benchmark 1, we found that the PSOGO-Senti model improved the two-polarity sentiment analysis by 5.131%. From the performance comparison between PSOGO-Senti and benchmark 5, we found that the PSOGO-Senti model improved the performance by 0.109%. From the performance comparison between PSOGO-Senti and benchmark 6, we found that the PSOGO-Senti model improved the performance by 9.631%. From the performance comparison between PSOGO-Senti and benchmark 4, we found that the PSOGO-Senti model improved the performance by 0.217%. The comparison of PSOGO-Senti and benchmark 2 revealed that the PSOGO-Senti model improved the performance by 2.558%. The comparison of PSOGO-Senti and benchmark 3 revealed that the PSOGO-Senti model improved the performance by 0.109%.

The performance of the three-polarity sentiment analysis is shown in Table 8. Several interesting findings were observed following this analysis. First, we observed that the PSOGO-Senti model achieved the best performance with an average precision of 0.759, average recall of 0.753 and F-measure of 0.755. Second, from the performance comparison between PSOGO-Senti and benchmark 7, we found that the PSOGO-Senti approach improved over the random IG subset dimension *k* and default SVM parameters *c* and γ approach by 5.447%. From the performance comparison between PSOGO-Senti and benchmark 1, we found that the PSOGO-Senti model improved the performance of the three-polarity sentiment analysis by 4.716%. From the performance comparison between PSOGO-Senti and benchmark 5, we found that the PSOGO-Senti model improved the performance by 2.165%. From the performance comparison between PSOGO-Senti and benchmark 6, we found that the PSOGO-Senti model improved the performance by 2.165%. From the performance comparison between PSOGO-Senti and benchmark 4, we found that the PSOGO-Senti model improved the performance by 1.206%. From the performance comparison between PSOGO-Senti and benchmark 2, we found that the PSOGO-Senti model improved the performance by 1.342%. From the performance comparison between PSOGO-Senti and benchmark 3, we found that the PSOGO-Senti model improved the performance by 0.936%.

**Table 8:** Performance Comparison of PSOGO-Senti and Benchmarks in the Three-Polarity Sentiment Analysis (Guahao Dataset).

	Precision	Recall	F-measure
**Benchmark 1**: Optimal *k* + Default *c* + Default γ	0.739	0.718	0.721
**Benchmark 2**: Optimal *k* + Default *c* + Optimal γ	0.752	0.743	0.745
**Benchmark 3**: Optimal *k* + Optimal *c* + Default γ	0.753	0.747	0.748
**Benchmark 4**: *k* = *5000* + Optimal *c* + Default γ	0.752	0.745	0.746
**Benchmark 5**: *k* = *5000* + Optimal *c* + Optimal γ	0.743	0.737	0.739
**Benchmark 6**: *k* = *5000* + Default *c* + Optimal γ	0.743	0.737	0.739
**Benchmark 7**: *k* = *5000* + Default *c* + Default γ	0.729	0.714	0.716
**PSOGO-Senti:**	0.759	0.753	0.755

The performance of the five-polarity sentiment analysis is detailed in Table 9. Several interesting findings were observed as a result of this analysis. First, we observed that the PSOGO-Senti model achieved the best performance with an average precision of 0.694, average recall of 0.690 and F-measure of 0.691. Second, from the performance comparison between PSOGO-Senti and benchmark 7, we found that the PSOGO-Senti approach improved over the random IG subset dimension *k* and default SVM parameters *c* and γ by 10.207%. From the performance comparison between PSOGO-Senti and benchmark 1, we found that the PSOGO-Senti model improved the performance of the five-polarity sentiment analysis by 17.119%. From the performance comparison between PSOGO-Senti and benchmark 5, we found that the PSOGO-Senti model improved the performance by 0.436%. From the performance comparison between PSOGO-Senti and benchmark 6, we found that the PSOGO-Senti model improved the performance by 4.223%. From the performance comparison between PSOGO-Senti and benchmark 4, we found that the PSOGO-Senti model improved the performance by 0.145%. From the performance comparison between PSOGO-Senti and benchmark 2, we found that the PSOGO-Senti model improved the performance by 2.675%. From the performance comparison between PSOGO-Senti and benchmark 3, we found that the PSOGO-Senti model improved the performance by 0.436%.

**Table 9:** Performance Comparison of PSOGO-Senti and Benchmarks in the Five-Polarity Sentiment Analysis (Guahao Dataset).

	Precision	Recall	F-measure
**Benchmark 1**: Optimal *k* + Default *c* + Default γ	0.637	0.587	0.590
**Benchmark 2**: Optimal *k* + Default *c* + Optimal γ	0.680	0.672	0.673
**Benchmark 3**: Optimal *k* + Optimal *c* + Default γ	0.690	0.688	0.688
**Benchmark 4**: *k* = *5000* + Optimal *c* + Default γ	0.690	0.690	0.690
**Benchmark 5**: *k* = *5000* + Optimal *c* + Optimal γ	0.690	0.687	0.688
**Benchmark 6**: *k* = *5000* + Default *c* + Optimal γ	0.674	0.661	0.663
**Benchmark 7**: *k* = *5000* + Default *c* + Default γ	0.647	0.624	0.627
**PSOGO-Senti**:	0.694	0.690	0.691

Based on the dataset of Guahao medical reviews, from the comparison between the feature subset optimized by PSOGO-Senti model and features in HowNet, we observed several interesting findings. First, we found that the features in the optimal feature subset selected by PSOGO-Senti is far fewer than those in HowNet. The results in Table 5 showed that there are 8742 features in HowNet. There are 2537 features in the optimal feature subset selected by PSOGO-Senti in the two-polarity sentiment analysis. In the three-polarity sentiment analysis, there are 762 features in the optimal feature subset. There are 1686 features in the optimal feature subset selected by PSOGO-Senti in the five-polarity sentiment analysis. The results indicated that PSOGO-Senti can effectively remove redundant and noisy features. Second, in the feature subset optimized by PSOGO-Senti, a high proportion of features are not included in the HowNet dictionary. The extracted unique features in the optimal feature subset are usually valuable, domain-specific and possess a high-explanatory power, such as “不耐烦” (impatient), “久” (for a long time), “耐心” (patient), “庸医” (quack), “敷衍了事” (do things carelessly), “不怎么样” (not very good), “草草了事” (go through a thing carelessly), “答非所问” (give an irrelevant answer), “名不符实” (undeserved reputation), and “救死扶伤” (heal the wounded and rescue the dying). In the two-polarity sentiment analysis, 2166 features are in the optimal feature subset of PSOGO-Senti, but not in the HowNet dictionary. That is to say, 85.38% of the features in the optimal feature subset are not included in HowNet. In three-polarity sentiment analysis, 624 features are in the optimal feature subset of PSOGO-Senti, but not in HowNet. That is to say, 81.89% of the features in the optimal feature subset are not included in HowNet. In five-polarity sentiment analysis, 1403 features are in the optimal feature subset of PSOGO-Senti, but not in HowNet. That is to say, 83.21% of the features in the optimal feature subset are not included in HowNet. Table 10 lists certain features that are in the optimal feature subset of PSOGO-Senti but not in the HowNet dictionary. This indicated that PSOGO-Senti can extract and select an optimal feature subset with a higher-explanatory power for medicine-specific sentiment analysis. Based on the optimal feature subset, PSOGO-Senti achieved satisfactory performance in medical sentiment analysis.

**Table 10:** Some of the Features in the Optimal Feature Subset of PSOGO-Senti but not in the HowNet Dictionary (Guahao Dataset).

	Features
Verb	浪费 (waste), 打发 (send away), 折腾 (cause physical or mental suffering), 不理 (ignore), 坑人 (harm), 无奈 (feel helpless)
Adjective	不耐烦 (impatient),久 (for a long time), 耐心 (patient),丰富 (experienced), 体贴 (considerate)
Noun	黄牛 (scalper), 庸医 (quack), 复查 (reexamination)
Phrase	敷衍了事 (do things carelessly), 不怎么样 (not very good), 草草了事 (go through a thing carelessly), 莫名其妙 (without rhyme or reason), 答非所问 (give an irrelevant answer), 名不符实(undeserved reputation), 救死扶伤 (heal the wounded and rescue the dying)

#### Experiment 3: Comparison experiments of PSOGO-Senti and GA-based, GSM-based approaches

The results from the proposed PSOGO-Senti model are compared with those from the GSM- and GA-based models. The experimental results are reported in Tables 11 and 12.

**Table 11:** Performance Comparison of PSOGO-Senti and the GSM-, GA-based Approaches (1).

Dataset	Ctrip Dataset								
Polarities	Two-polarity			Three-polarity			Five-polarity		
Approach	PSOGO-Senti	GA	GSM	PSOGO-Senti	GA	GSM	PSOGO-Senti	GA	GSM
Precision	0.906	0.915	0.905	0.695	0.737	0.690	0.521	0.500	0.467
Recall	0.906	0.915	0.905	0.696	0.747	0.690	0.522	0.485	0.467
F-measure	0.906	0.915	0.905	0.695	0.741	0.690	0.519	0.484	0.465
Optimal *k*	1120	1930	1001	1074	3157	1261	5000	2882	501

**Table 12:** Performance Comparison of PSOGO-Senti and the GSM-, GA-based Approaches (2).

Dataset	Guahao Dataset								
Polarities	Two-polarity			Three-polarity			Five-polarity		
Approach	PSOGO-Senti	GA	GSM	PSOGO-Senti	GA	GSM	PSOGO-Senti	GA	GSM
Precision	0.922	0.925	0.922	0.759	0.753	0.743	0.694	0.689	0.670
Recall	0.922	0.925	0.922	0.753	0.750	0.742	0.690	0.686	0.666
F-measure	0.922	0.925	0.922	0.755	0.751	0.742	0.691	0.686	0.666
Optimal *k*	2537	3323	4161	762	3239	1261	1686	4119	461

Based on both datasets, from the performance comparison between the PSOGO-Senti model and GSM-based model, we found that the performance of PSOGO-Senti surpassed that of GSM. Moreover, comparing the improvements obtained for the two-polarity, three-polarity and five-polarity sentiment analysis results, we found higher improvements of the PSOGO-Senti than those of GSM for more sentimental polarities. For example, in the two-polarity sentiment analysis, based on both datasets, PSOGO-Senti was found to deliver comparable results relative to GSM. However, in the five-polarity sentiment analysis, PSOGO-Senti improved the performance by 3.754% compared with that of GSM based on the dataset of Guahao medical reviews. Based on the dataset of Ctrip tourism reviews, in the five-polarity sentiment analysis, PSOGO-Senti improved the performance by 11.613% compared with that of GSM.

From the performance comparison between the PSOGO-Senti model and GA-based model, we found that PSOGO-Senti achieved similar performance to the GA-based approach in the two-polarity sentiment analysis. However, in the five-polarity sentiment analysis, the performance of PSOGO-Senti was better than that of the GA-based approach. For example, based on the dataset of Ctrip tourism reviews, in the two-polarity sentiment analysis, GA was found to deliver comparable results relative to PSOGO-Senti. However, in the five-polarity sentiment analysis, PSOGO-Senti improved the performance by 7.231% compared with GA. The above experimental results demonstrate that PSOGO-Senti is more suitable for improving a difficult multi-polarity sentiment analysis.

## Discussion

The following observations were made from the experimental results. First, a global optimization approach in sentiment classification can improve the performance of two-, three- and five-polarity Chinese sentiment analyses. Second, the performance comparison demonstrated that global optimization parameters exist and that the selection of feature dimension *k* is related to the SVM parameters *c* and *γ*. The PSOGO-Senti model achieves optimal performance when the feature dimension *k* and SVM parameters *c* and *γ* are all optimized. Third, the PSOGO-Senti model achieves satisfactory sentiment analysis performance on both the Ctrip tourism dataset and Guahao medical dataset. This indicates that PSOGO-Senti is an effective and robust sentiment analysis method. Fourth, from the improvement comparison of the two-polarity, three-polarity and five-polarity sentiment analysis results, we found that the five-polarity sentiment analysis delivered the largest improvement. The improvement of the two-polarity sentiment analysis was the smallest. Based on the dataset of Ctrip tourism reviews, PSOGO-Senti improved over the random IG subset dimension *k* and default SVM parameters *c* and γ by 4.138% in the two-polarity sentiment analysis. In the three-polarity sentiment analysis, PSOGO-Senti improved over the random IG subset dimension *k* and default SVM parameters *c* and γ by 6.595%. In the five-polarity sentiment analysis, PSOGO-Senti improved over the random IG subset dimension *k* and default SVM parameters *c* and γ by 14.066%. Based on the dataset of Guahao medical reviews, PSOGO-Senti improved over the random IG subset dimension *k* and default SVM parameters *c* and γ by 2.673% in the two-polarity sentiment analysis. In the three-polarity sentiment analysis, PSOGO-Senti improved over the random IG subset dimension *k* and default SVM parameters *c* and γ by 5.447%. In the five-polarity sentiment analysis, PSOGO-Senti improved over the random IG subset dimension *k* and default SVM parameters *c* and γ by 10.207%. Therefore, we can conclude that the PSOGO-Senti approach achieves higher improvement for a more complicated sentimental analysis task. Fifth, from the comparison between the optimal feature subset selected by PSOGO-Senti and the features in the HowNet dictionary, we observed that the features in the optimal feature subset selected by PSOGO-Senti were far fewer than those in HowNet. Moreover, many domain-specific features with a higher-explanatory power in the optimal feature subset were not included in the HowNet dictionary. From the results of experiments 1 and 2, based on both datasets, more than 80% of the features in the PSOGO-Senti optimal feature subset were not included in the HowNet dictionary. The results indicate that PSOGO-Senti can remove redundant and noisy features and can select an optimal feature subset with a higher-explanatory power for domain-specific sentiment analysis. Sixth, from the performance comparison of the PSOGO-Senti, GA-based and GSM-based approaches, we found that the performances of PSOGO-Senti and GA surpass the performance of GSM. Moreover, based on both datasets, comparing the improvements obtained for the two-polarity, three-polarity and five-polarity sentiment analysis results, we found higher improvements of the PSOGO-Senti than those of GSM for more sentimental polarities. From the performance comparison between PSOGO-Senti and the GA-based approach, we found that PSOGO-Senti achieved a similar performance to that of the GA-based approach in the two-polarity sentiment analysis. However, in the five-polarity sentiment analysis, the performance of PSOGO-Senti was better than that of the GA-based approach. Therefore, we conclude that PSOGO-Senti is more suitable for improving a difficult multi-polarity sentiment analysis problem.

## Conclusion and Future Studies

Sentiment analysis has many useful applications in business. In this study, we proposed a global optimization-based (PSOGO-Senti) approach for performing a sentiment analysis task with IG for feature selection and an SVM as the learning engine. The PSOGO-Senti approach utilizes a particle swarm optimization algorithm to obtain a global optimal combination of the feature subset and SVM parameters *c* and γ. Our study yields the following contributions. First, IG has been identified as an effective method of sentimental term extraction [7–10]; however, little emphasis has been placed on the optimal feature subset selection for a particular sentiment analysis task [7]. The SVM is the most popular sentiment classification algorithm; however, if the parameters are not set well, the results may turn out to be unsatisfactory. Moreover, the sentimental feature subset choice influences the appropriate kernel parameters, and vice versa [24]. Therefore, a global optimization strategy is needed for sentimental feature subset and SVM parameter selection. However, in sentiment analysis research, little emphasis has been focused on the global optimization approach for the selection of an optimal feature subset and associated parameters. At present, the effectiveness of applying a global optimization approach to sentiment analysis remains unclear. Our experimental results show that our proposed PSOGO-Senti achieves the best performance when the feature dimension *k* and SVM parameters *c* and *γ* are all optimized. We conclude that the PSOGO-Senti approach can improve the performance of two-, three- and five-polarity Chinese sentiment analyses. Second, the PSOGO-Senti approach is applied to the sentiment analysis on both tourist reviews and medical reviews. Two experimental results showed that the PSOGO-Senti approach was effective and robust for sentiment analysis tasks in different domains. Third, from the improvement comparison of two-polarity, three-polarity and five-polarity sentiment analysis, we found that the five-polarity sentiment analysis delivered the largest improvement. The improvement of the two-polarity sentiment analysis was the smallest. Therefore, we conclude that the PSOGO-Senti approach achieves higher improvement for a more complicated sentiment analysis task. Fourth, from the comparison between the optimal feature subset selected by PSOGO-Senti and the sentimental dictionary, we found PSOGO-Senti can remove redundant and noisy features and can select an optimal feature subset with a higher-explanatory power when used for domain-specific sentiment analysis. Fifth, from the performance comparison of the PSOGO-Senti, GSM- and GA-based approaches, we found that PSOGO-Senti is more suitable for improving a difficult multi-polarity sentiment analysis problem.

In future studies, we will apply our sentiment analysis model to other domains, such as other types of user reviews and news articles. We aim to apply the PSOGO-Senti model to multiple languages in addition to Chinese. We also intend to investigate the parameter optimization process through more efficient methods.
